# Commensal Bacteria-Dependent CD8αβ^+^ T Cells in the Intestinal Epithelium Produce Antimicrobial Peptides

**DOI:** 10.3389/fimmu.2018.01065

**Published:** 2018-05-16

**Authors:** Banru Chen, Xiang Ni, Rui Sun, Benhua Zeng, Hong Wei, Zhigang Tian, Haiming Wei

**Affiliations:** ^1^The CAS Key Laboratory of Innate Immunity and Chronic Disease, School of Life Sciences and Medical Center, Institute of Immunology, University of Science and Technology of China, Hefei City, Anhui, China; ^2^Hefei National Laboratory for Physical Sciences at Microscale, University of Science and Technology of China, Hefei City, Anhui, China; ^3^Department of Laboratory Animal Science, College of Basic Medical Sciences, Army Medical University, Chongqing, China

**Keywords:** CD8αβ^+^ intraepithelial lymphocytes, α-defensins, antimicrobial activity, intestinal epithelium, commensal bacteria

## Abstract

The epithelium of the intestine functions as the primary “frontline” physical barrier for protection from enteric microbiota. Intraepithelial lymphocytes (IELs) distributed along the intestinal epithelium are predominantly CD8^+^ T cells, among which CD8αβ^+^ IELs are a large population. In this investigation, the proportion and absolute number of CD8αβ^+^ IELs decreased significantly in antibiotic-treated and germ-free mice. Moreover, the number of CD8αβ^+^ IELs was correlated closely with the load of commensal microbes, and induced by specific members of commensal bacteria. Microarray analysis revealed that CD8αβ^+^ IELs expressed a series of genes encoding potent antimicrobial peptides (AMPs), whereas CD8αβ^+^ splenocytes did not. The antimicrobial activity of CD8αβ^+^ IELs was confirmed by an antimicrobial-activity assay. In conclusion, microbicidal CD8αβ^+^ IELs are regulated by commensal bacteria which, in turn, secrete AMPs that have a vital role in maintaining the homeostasis of the small intestine.

## Introduction

Intestinal epithelial cells (IECs) are a single layer of cells covering the luminal side of the intestinal tract. These cells are confronted by trillions of resident commensal bacteria (1). Intraepithelial lymphocytes (IELs) are the lymphocytes located between enterocytes. IELs have a vital role in protective immunity against invading pathogens, as well as providing tolerance to commensal bacteria, and maintaining intestinal homeostasis (2–4). These “frontline” IELs are exclusively T cells and are predominantly CD8^+^ and can be divided into two subsets based on the expression of T cell receptors and co-receptors (3). Among the two subsets, type A IELs are conventional CD8αβ^+^TCRαβ^+^ or CD4^+^TCRαβ^+^ cells and are pre-activated in gut-associated lymphoid tissues or intestinal draining lymph nodes (LNs), before they “home” to the intestinal epithelium. These T cells show an “effector-memory-like phenotype” and have a protective role against various pathogens. Type B IELs are CD8αα^+^ TCRαβ^+^ or CD8αα^+^ TCRγδ^+^ and do not express CD4 or CD8αβ. These T cells participate in damage repair, the regulatory response, and are potent cytotoxic effectors (2, 5–12).

CD8αβ^+^ IELs are the major type A IELs. They are the progeny of conventional CD8 T cells activated by peripheral antigens in Peyer’s patches (PP) or mesenteric LNs and accumulate with age in the epithelium of mice (2, 13). Adoptive transfer of antigen-specific CD8αβ^+^ IELs into mice infected with lymphocytic choriomeningitis virus, rotavirus, *Giardia lamblia*, or *Toxoplasma gondii* has demonstrated a protective function for CD8αβ^+^ IELs in infection (14–17). CD8αβ^+^ IELs are strongly cytotoxic, and are a subset of antigen-experienced cytotoxic T lymphocytes (CTLs) (2). However, these IELs typically encounter a large number of commensal bacteria rather than invading pathogens, so two main questions arise: what is the effect of CD8αβ^+^ IELs on commensal bacteria? Do CD8αβ^+^ IELs function in ways other than conventional CTLs when confronted by commensal bacteria? These questions and the relationship of these IELs to commensal bacteria have not been explored fully. Comparison of wild-type, germ-free (GF), and antibiotic-treated mice has demonstrated a positive relationship between CD8αβ^+^ IELs and commensal bacteria, suggesting that the latter may regulate the number of CD8αβ^+^ IELs in the intestinal epithelium (18–20). However, the mechanistic basis for the effect of CD8αβ^+^ IELs on commensal bacteria is not known.

As a physical barrier between the connective tissue and intestinal lumen, the intestinal epithelium utilizes various antibacterial mechanisms to maintain a steady-state relationship between the host and bacteria. Defensins within intestinal crypts have a critical role in this relationship. Defensins are a family of antimicrobial peptides (AMPs) that can be divided into three subfamilies α-, β-, and θ-defensins, according to their structural features (21). In humans and other mammals, α-defensins are usually secreted by neutrophils, monocytes, and some epithelial cells. In mice, leukocytes do not express α-defensins but Paneth cells do, and are located in the crypts of the small intestine, where α-defensins are a predominant antimicrobial factor (21–24). Paneth cells are located at the base of crypts (which are at the bottom of villi) and release AMPs in the form of secretory granules (3). Villi extend into the lumen, and have a large surface area for contact with intestinal contents. Whether α-defensins are produced along the surface of villi is not known. However, it is possible that some lymphocytes scattered along the intestinal epithelium may also produce anti-microbial factors that help to control the large number of microorganisms within the lumen.

To study the relationship between CD8αβ^+^ IELs and commensal bacteria, we examined CD8αβ^+^ IELs in GF and antibiotic-treated mice and showed that they are dependent upon commensal bacteria. In an experiment involving microbiota transplantation, we provide evidence that CD8αβ^+^ IELs can be induced by some specific members of commensal bacteria. In addition, a similar decrease in the number of CD8αβ^+^ IELs in toll-like receptor (TLR)-deficient mice suggested commensal bacteria may regulate the number of CD8αβ^+^ IELs *via* TLR stimulation. We conducted microarray analysis and showed that CD8αβ^+^ IELs expressed a series of α-defensins at gene and protein levels. The supernatants of cultured CD8αβ^+^ IELs killed bacteria directly and showed antimicrobial activity. Overall, our results provide evidence for a new function by which CD8αβ^+^ IELs regulate commensal bacteria in the epithelium of the small intestine.

## Materials and Methods

### Mice

Wild-type [specific pathogen-free (SPF) and GF] mice on a C57BL/6 background were purchased from the Shanghai Laboratory Animal Center (SLAC, Chinese Academy of Sciences). GF and corresponding SPF mice on a BALB/c background were generated and provided by Professor Hong Wei from Third Military Medical University (Chongqing, China). TLR2^−/−^, TLR4^−/−^, TLR9^−/−^, and TLR2^−/−^TLR4^−/−^TLR9^−/−^ mice (SPF) on a C57BL/6 background were a gift from Dr. Shaobo Su (Sun Yat-sen University, Guangzhou, China). GF mice were housed in GF facilities according to animal care regulations. SPF mice were maintained in a SPF barrier facility at the University of Science and Technology of China (USTC, Hefei, China) in accordance with the guidelines for use of experimental animals. Sex-matched mice (8–10 weeks) were used for all experiments unless indicated otherwise.

### Isolation of IELs and Splenocytes (SPLs)

Intraepithelial lymphocytes were isolated as described previously (25) with modifications. The entire small intestines were extracted and placed in cold phosphate-buffered saline (PBS) after mesenteric fat tissue had been removed. After careful identification of individual PPs on the anti-mesenteric side of the intestinal serosa and excising them using surgical scissors, the small intestines were opened longitudinally and washed with PBS 4–5 times to remove most of the contents. Then, the intestines were cut into pieces (1 cm) and placed in a 50-mL Erlenmeyer flask. The intestinal pieces were incubated in 20 mL of Iscove’s modified Dulbecco’s medium (IMDM) supplemented with 5% fetal bovine serum (FBS), 5 mM ethylenediamine tetra-acetic acid (EDTA), and 15 mM HEPES, and rotated at 200 rpm for 30 min at 37°C. This procedure was repeated thrice. After each rotation, the supernatants containing IELs were collected, and new IMDM with EDTA and HEPES was added to the Erlenmeyer flask. The supernatants were filtered through a 200-gauge steel mesh and cells (including IELs) were collected by centrifugation. Total cells were resuspended in 3 mL of 40% Percoll and then overlaid onto 2 mL of 70% Percoll, after which gradient centrifugation was undertaken at room temperature. IELs at the interphase were collected. The cells were washed once in PBS and these IELs were used for experimentation.

Spleens were extracted and placed in cold PBS. After filtration through a 200-gauge steel mesh and the removal of red blood cells (RBC) with RBC lysis buffer, SPLs were collected for further experimentation.

### Flow Cytometry and Sorting

For surface staining, IELs and SPLs were incubated with 5% normal rat serum to block FcRs and incubated with indicated antibodies for 30 min at 4°C. For intracellular staining, IELs and SPLs were pretreated with phorbol myristate acetate (PMA; 50 ng/mL; Sigma–Aldrich, Saint Louis, MO, USA), ionomycin (1 µg/mL; Sigma–Aldrich), and monensin (10 µg/mL; Sigma–Aldrich) for 5 h. Then, the stimulated IELs and SPLs were incubated with 5% normal rat serum to block FcRs and stained with surface markers. After fixation and permeabilization, cells were stained further with intracellular antibody. The antibodies used are listed in Table S1 in Supplementary Material. All flow-cytometry experiments were carried out with a flow cytometer (LSRII; BD Biosciences, Franklin Lakes, NJ, USA) and analyzed with FlowJo software (Tree Star, Ashland, OR, USA). For microarray analysis, sorting of CD8αβ^+^ IELs and CD8αβ^+^ SPLs was undertaken with a FACS Aria cell sorter (BD Biosciences) and purity >98% was achieved.

We carried out an assay to measure antimicrobial activity. To obtain cells with increased activity, purified CD8αβ^+^ IELs and CD8αβ^+^ SPLs were sorted by magnetic-activated cell sorting (MACS; Miltenyi Biotec, Bergisch Gladbach, Germany). The cells were labeled with biotin anti-mouse CD8b antibody and then anti-biotin microbeads. The purity of the sorted cell populations was >90%.

### Antibiotic Treatment

Commensal bacteria from the gut were depleted using four antibiotics according to method reported before (26) with modifications. Briefly, for combinatorial antibiotic treatment, mice were provided with sterile water supplemented with metronidazole (1 g/L), neomycin sulfate (1 g/L), ampicillin (1 g/L), and vancomycin (0.5 g/L) (Sangon Biotech, Shanghai, China) beginning at 3 weeks of age and lasting for 5 weeks. For single antibiotic treatment, mice were treated with sterile water supplemented with each antibiotic alone at the dose indicated above. Water containing antibiotics was supplied as drinking water to mice and changed twice a week.

### Analyses of Bacterial 16S rDNA

The contents of a 15-cm distal portion of small intestines were collected and weighed. Bacterial DNA was extracted using an E.Z.N.A™ Mag-Bind Soil DNA kit (Omega Bio-Tek, Norcross, GA, USA). To measure the composition of bacteria in the small intestine, the V3–V4 region of 16S rDNA was amplified and sequenced using Illumina Miseq™ (Sangon Biotech).

### Transplantation With *Bifidobacterium*

The transplantation experiment was carried out according to previous reports with modifications (27, 28). Mice at 3 weeks of age were treated with a combination of metronidazole (1 g/L), neomycin sulfate (1 g/L), ampicillin (1 g/L), and vancomycin (0.5 g/L) (Sangon Biotech) for 2 weeks. Then, at 5 weeks of age, the water was replaced by antibiotic-free water and the mice were administered (i.g.) with a mixture of 2 × 10^8^ CFU *Bifidobacterium longum* (ATCC15697) and 2 × 10^8^ CFU *Bifidobacterium adolescentis* (ATCC15703). Also, the antibiotic-free drinking water was supplied with *B. longum* (final concentration ≈10^6^ CFU/mL) and *B. adolescentis* (final concentration ≈10^6^ CFU/mL). Mice in the control group were administered (i.g.) with an equal volume of Modified Reinforced Clostridial Medium (BD 218081) but without bacterial cells and the antibiotic-free drinking water was also supplied with an equal volume of Modified Reinforced Clostridial Medium. Mice were evaluated after 15 days.

### Identification of Bacteria

Nine days after transplantation of *Bifidobacterium*, fresh stool samples were collected and weighed. To identify the existence and relative expression of *Bifidobacterium*, DNA from the stool samples was extracted using a Stool Genome DNA Extraction kit (YPH-Bio, Beijing, China) according to manufacturer instructions. The abundance of *Bifidobacterium* was analyzed by quantitative real-time polymerase chain reaction (qRT-PCR) with SYBR^®^ Premix Ex Taq (Tli RNaseH Plus; TaKaRa Bio, Kusatsu, Japan). The relative expression of the target gene was normalized to the gene level of “all bacteria.”

As reported previously (29, 30), the following primers (forward and reverse, respectively) were used: “all bacteria”: 5′-CGGTGAATACGTTCCCGG-3′ and 5′-TACGGCTACCTTGTTACGACTT-3′; *Bifidobacterium*: 5′-CTCCTGGAAACGGGTGG-3′ and 5′-GGTGTTCTTCCCGATATCTACA-3′.

### Microarray Analysis

Samples were collected from purified CD8αβ^+^ IELs and CD8αβ^+^ SPLs, with ≈1 million cells in each sample. Microarray analysis was done with PrimeView™ chips (Affymetrix, Santa Clara, CA, USA) and analyzed using MeV 4.9 software.

### Real-Time Polymerase Chain Reaction

RNA purified from 1 million CD8αβ^+^ IELs and 1 million CD8αβ^+^ SPLs was isolated using TRIzol™ Reagent (Invitrogen, Carlsbad, CA, USA). Primers were synthesized by Sangon and are listed in Table S2 in Supplementary Material.

### Immunofluorescence

Isolated CD8^+^ IELs were attached to poly-l-lysine-coated slides and then fixed for 20 min with 4% paraformaldehyde at room temperature, after which the slides attached with CD8^+^ IELs were stored at −80°C. Slides with IELs were moved to room temperature for 5 min and permeabilized with 1% Triton X-100 for 15–20 min, followed by blocking with 1% bovine serum albumin for 1 h at room temperature. Subsequently, slides were stained with primary antibodies at 4°C overnight. The slides were then washed thrice in PBST (PBS with 0.05% Tween 20) for 5 min. Slides were incubated with secondary antibody in the dark for 2 h at 37°C and then stained with 4′,6-diamidino-2-phenylindole (1 ng/mL) for 3 min at room temperature. After a final wash in PBST, the slides were mounted on coverslips. Cells were imaged with a microscope (LS710; Zeiss, Wetzlar, Germany). Detailed information about antibodies is listed in Table S1 in Supplementary Material.

### Stimulation of Secretion of CD8αβ^+^ T Cells

CD8αβ^+^ IELs (5 × 10^5^) and CD8αβ^+^ SPLs (5 × 10^5^) purified by MACS were cultured in IMDM (1 mL) supplemented with 5% FBS for 24 h at 37°C. During this 24 h, PMA (50 ng/mL, Sigma–Aldrich) and ionomycin (1 µg/mL, Sigma–Aldrich) were added during the last 6 h, or an interleukin (IL)-15/IL-15R complex (50 ng/mL) was added during the entire 24-h period. IMDM with 5% FBS and streptomycin (0.1 mg/mL) was used as a positive control because the antibiotic streptomycin is known to inhibit *Escherichia coli*. After 24 h, cellular components were deposited by centrifugation, and supernatants were collected for an assay to measure antimicrobial activity.

### Assay to Measure Antimicrobial Activity

*Escherichia coli* [a gift from Dr. Shujuan Lv (Anhui Medical University, Anhui, China)] was cultivated aerobically in liquid Luria Bertani (LB) medium at 37°C for 8 h and the concentration was diluted to 1 × 10^5^ CFU/mL. LB-Agar Medium petri plates were coated with *E. coli* (1 × 10^5^ CFU/mL) and then sterile Oxford cups (8 mm in diameter) were placed on the surface of the each bacteria-inoculated agar plate. Next, supernatants (200 µL) from stimulated CD8αβ^+^ IELs or CD8αβ^+^ SPLs were dropped into the corresponding Oxford cups, and equivalent amounts of IMDM with streptomycin were used as positive controls. The plates were incubated for 18 h at 37°C. The antimicrobial activity was shown by the clear inhibition zone (called “inhibition ring”) around the sample-loaded Oxford cups after incubation and the diameter of the inhibition rings were measured.

### Statistical Analyses

The unpaired Student’s *t*-test was used for two groups. A normal distribution was evaluated by the Kolmogorov–Smirnov test. If data showed a normal distribution, they were tested by an unpaired *t*-test (parametric test) but, if not, the Mann–Whitney test (nonparametric test) was used. A one-way ANOVA for more than two groups were used to determine significant differences. Data were presented as mean ± SEM. *P* < 0.05 was considered significant.

### Data Availability Statements

Microarray data have been deposited at the National Center for Biotechnology Information GEO repository through accession number GSE105061.

## Results

### Commensal Bacteria Specifically Regulate the Number of CD8αβ^+^ IELs in the Intestinal Epithelium

**Figure 1:**
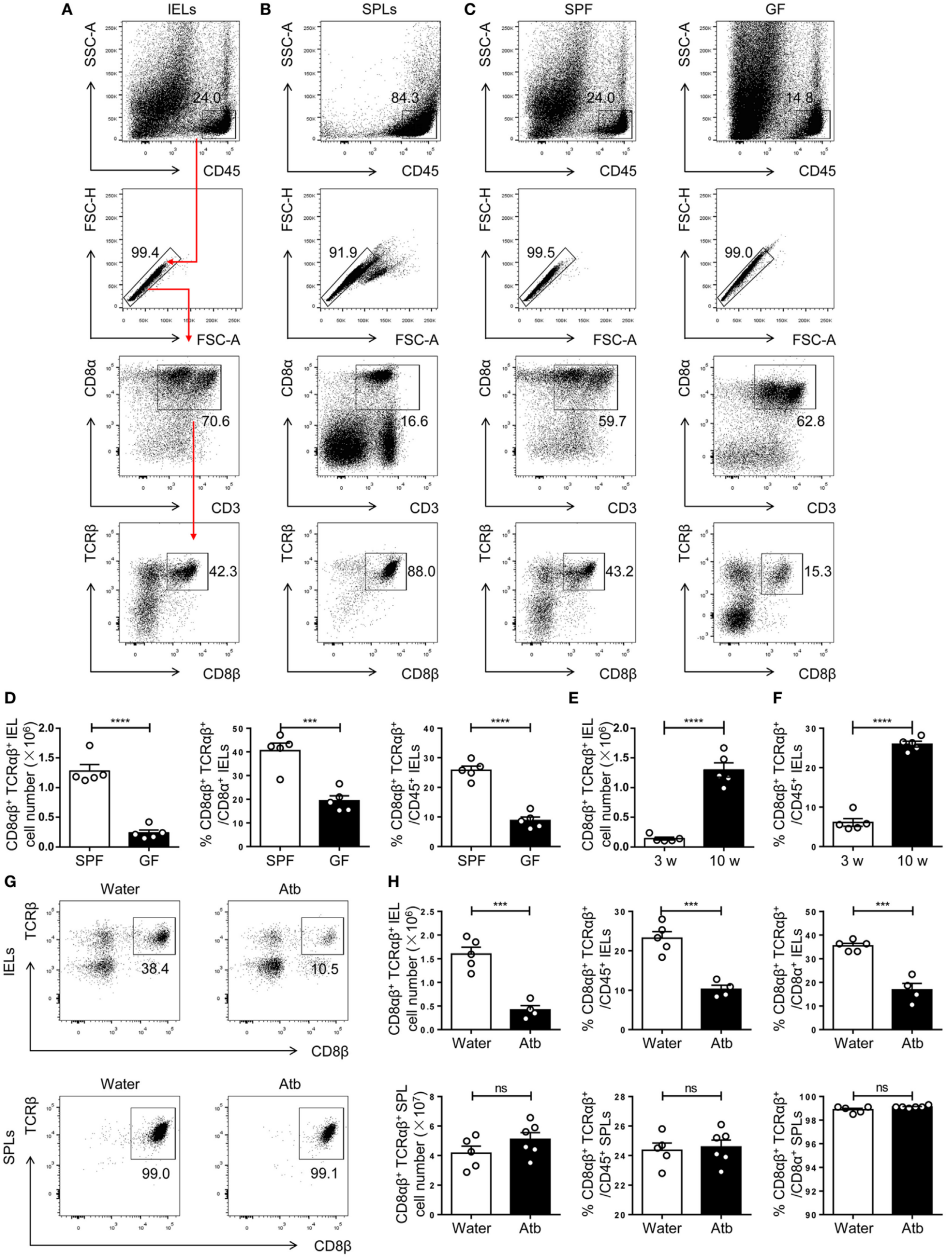
Commensal bacteria direct the number and frequency of CD8αβ^+^ IELs in the epithelium of the small intestine. **(A,B)** Gating strategy and representative flow spots of CD3^+^ CD8α^+^ CD8β^+^ TCRβ^+^ among CD45^+^ single cells from SPF mice. Flow cytometry was undertaken on IELs **(A)** and SPLs **(B)**. Red arrow in **(A)** indicates the order for gating. **(C)** CD8αβ^+^ IELs from SPF and germ-free (GF) mice on a C57BL/6 background were gated for further analyses by the strategy shown in **(A)**. **(D)** The absolute number and percentage of CD8αβ^+^ IELs were compared between SPF and GF mice. **(E,F)** The absolute number **(E)** and percentage **(F)** of CD8αβ^+^ IELs were compared between 3- and 10-week-old WT mice. **(G,H)** Three-week-old SPF mice were fed with normal water or water containing antibiotics for 5 weeks and identified, respectively, as water-treated mice and Atb-treated mice. The absolute number and percentage of CD8αβ^+^ IELs and SPLs from the indicated mice were analyzed by flow cytometry, *n* = 4–6 mice per group. Numbers adjacent to outlined areas denote the percentage of gated cells. All mice used were on a C57BL/6 background. Data are representative of two independent experiments. Unpaired *t*-test. Error bars represent the mean ± SEM. ***P < 0.001, ****P < 0.0001. Abbreviations: ns, not significantly different; IEL, intraepithelial lymphocyte; SPL, splenocyte; Atb, antibiotic; WT, wild-type; SPF, specific pathogen-free; GF, germ-free.

CD8αβ^+^ IELs (type A) are a population of resident effector memory T cells scattered along the epithelium of the small intestine (5). The commensal microbiota has an important role in the maintenance of immune homeostasis (31–34). Studies in GF mice have demonstrated a positive relationship between CD8αβ^+^ IELs and commensal bacteria within the small intestine (18–20, 31, 35), yet a systematic classification and comparison with other CD8αβ^+^ cells have not been made. To investigate further the crosstalk between CD8αβ^+^ IELs and commensal bacteria, we first verified the positive relationship between them. By flow cytometry, we gated CD45^+^ CD3^+^ CD8β^+^ CD8α^+^ TCRβ^+^ cells in the epithelium of the small intestine, as well as CD8αβ^+^ SPLs as a phenotypic comparison (Figures 1A,B). CD8αβ^+^ IELs comprised most intraepithelial T cells in SPF mice. A dramatic decrease in absolute numbers and proportions was shown in GF mice, including the proportion of CD8αβ^+^ IELs in all CD45^+^ lymphocytes and CD8^+^ T cells (Figures 1C,D). These results confirmed a role for commensal bacteria in the accumulation of CD8αβ^+^ IELs.

As mice age, commensal bacteria accumulate gradually in the intestine (36). The number of CD8αβ^+^ IELs in adult SPF mice was greater than that in newly weaned mice (Figures 1E,F). To mimic this condition, mice were treated with a combination of antibiotics beginning at 3 weeks of age and lasting for 5 weeks (Atb-treated mice). These mice were compared with mice that received water without antibiotics. A decrease in the number of CD8αβ^+^ IELs was observed in Atb-treated mice but not in mice treated with normal water. The number and proportion of CD8αβ^+^ SPLs were unchanged in Atb-treated mice (Figures 1G,H). These data demonstrated that commensal bacteria could regulate the number of CD8αβ^+^ IELs in the epithelium of the small intestine.

### CD8αβ^+^ IELs Are Induced by Specific Members of Commensal Microbes

**Figure 2:**
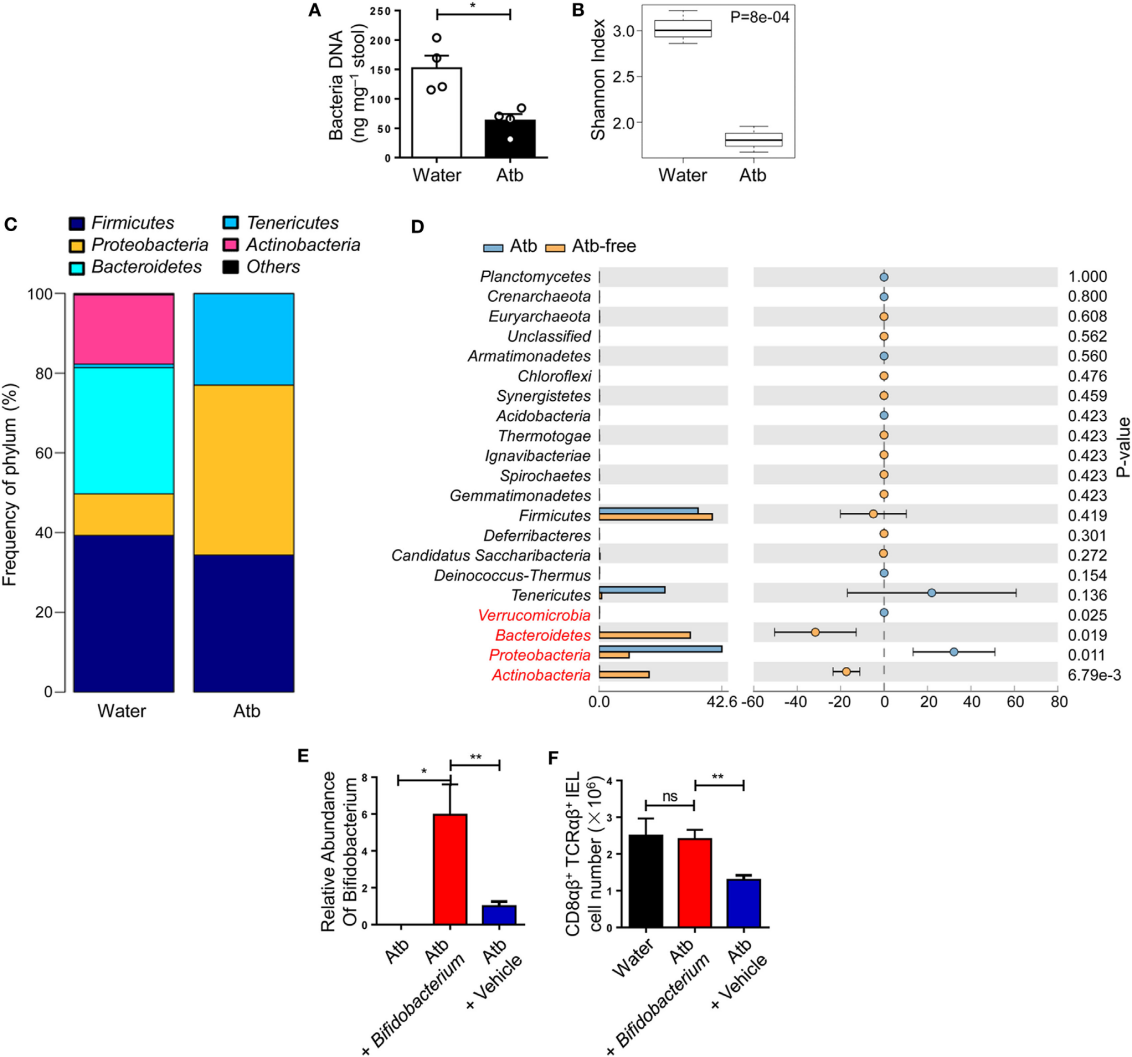
Global diversity of commensal bacteria affects the quantity of CD8αβ^+^ IELs. **(A–D)** Three-week-old mice were treated with normal water or water containing a combination of antibiotics (metronidazole, neomycin, ampicillin, and vancomycin) for 5 weeks. Analyses of 16S rDNA were done on bacterial DNA in the contents of the distal small intestine. **(A)** DNA load of bacteria in feces were detected (2 weeks post-treatment). **(B)** Plot of diversity using the Shannon Index. The Shannon Index of the two groups is shown to indicate the diversity of species. **(C)** Relative percentages of the dominant phylum in water-treated and Atb-treated mice were evaluated by the mean reads. **(D)** Distribution of intestinal bacterial groups in water-treated versus Atb-treated mice. The left portion indicates the proportion of the variety of bacteria phyla in water-treated and Atb-treated mice. The right portion indicates the difference in mean proportions between Atb-treated and water-treated groups, along with *P*-values and confidence intervals. **(E)** Quantitative PCR of bacterial DNA from fresh stool samples of mice pretreated with antibiotics for 2 weeks (left column); Atb-pretreated mice administered (i.g.) with *Bifidobacterium* (middle column) or vehicle (right column) (9 days post-transfer). Data show the relative abundance of *Bifidobacterium* in each group. **(F)** The absolute number of CD8αβ^+^ TCRαβ^+^ IELs was detected (15 days post-transfer). Data from mice treated with normal water from birth (left column), Atb-pretreated mice administered (i.g.) with *Bifidobacterium* (middle column), or vehicle (right column) (15 days post-transfer) are shown. Data are representative of two independent experiments. Unpaired *t*-test. Error bars represent the mean ± SEM. *P < 0.05, **P < 0.01. Abbreviations: ns, not significantly different; Atb, antibiotic; IEL, intraepithelial lymphocyte.

The number of CD8αβ^+^ IELs was affected by commensal bacteria, so the load and diversity of bacteria was analyzed in the intestine of Atb-treated mice. A sharp decrease in the load of commensal bacteria was observed after antibiotic treatment, as assessed by the difference in global microbe DNA loads between Atb-treated and water-treated mice (Figure 2A). This observation suggested that accumulation of CD8αβ^+^ IELs was influenced by the load of commensal microbes. Meanwhile, a decrease in the diversity in Atb-treated mice was shown by the Shannon index (Figure 2B). Moreover, the intestines of Atb-treated mice had a markedly different microbial composition, especially in the proportion of *Bacteroidetes* and *Actinobacteria* groups (Figure 2C). Antibiotic treatment essentially eliminated *Bacteroidetes* and *Actinobacteria*, which were predominant in mice that did not receive antibiotics (Figure 2D). Notably, the genera of *Bacteroidetes* and *Actinobacteria* disappeared completely in Atb-treated mice, whereas the genera within other phyla (e.g., *Fimicutes* and *Tenericutes*) were partially preserved (Figure S1A in Supplementary Material). Individual antibiotic treatment also revealed that CD8αβ^+^ IELs were influenced by some bacteria sensitive to ampicillin (Figures S1B,C in Supplementary Material).

To elucidate whether the gut microbiota eliminated by antibiotics, such as *Bifidobacterium* in phylum *Bacteroidetes*, could induce CD8αβ^+^ IELs, we carried out microbiota transplantation. After initial antibiotic treatment, *Bifidobacterium* species were removed completely (Figure 2E). Then, *Bifidobacterium* species were transferred to these mice by gavage and, 9 days later, the appearance of *Bifidobacterium* species was confirmed by qPCR of fecal material (Figure 2F). Colonization by *Bifidobacterium* species induced a robust increase in the number of CD8αβ^+^ IELs after 15 days (Figure 2F). Therefore, we concluded that *Bifidobacterium* species were members of the commensal microbiota that induced accumulation of CD8αβ^+^ IELs in the small intestine.

### TLR Signaling Is Required for Commensal Bacteria-Dependent CD8αβ^+^ IELs

A close relationship between commensal bacteria and CD8αβ^+^ IELs was demonstrated, then the regulation of CD8αβ^+^ IELs was explored. TLRs are expressed by macrophages, dendritic cells (DCs), and IECs, and recognize various commensal- and pathogen-associated molecular patterns (37, 38). To evaluate the impact of TLR signaling on type A IELs, CD8αβ^+^ IELs in various TLR-deficient mice were assessed.

**Figure 3:**
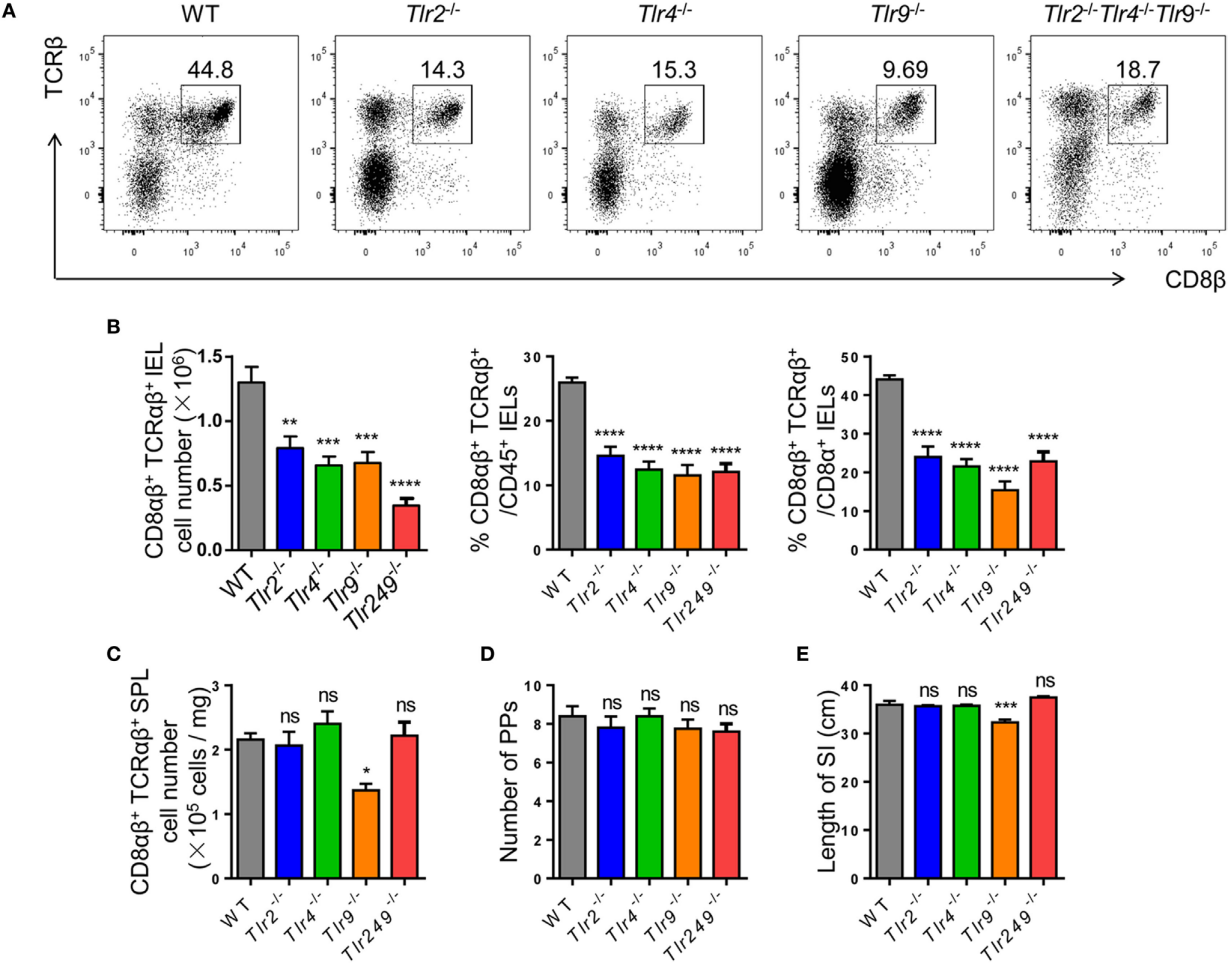
Commensal bacteria regulate the number of CD8αβ^+^ IELs *via* TLR (TLRs). **(A,B)** CD8αβ^+^ IELs from WT, TLR2^−/−^, TLR4^−/−^, TLR9^−/−^, and TLR2^−/−^TLR4^−/−^TLR9^−/−^ mice were gated by the strategy shown in Figure 1A **(A)**. Absolute number and percentage of CD8αβ^+^ IELs from indicated mice were calculated **(B)**. **(C–E)** The absolute number of CD8αβ^+^ splenocytes **(C)**, number of PP’s scattered along the small intestine **(D)**, and length of the entire small intestine **(E)** in indicated mice were counted and compared, *n* = 4–5 mice per group. All mice used were on a C57BL/6 background. Data are representative of three independent experiments. One-way ANOVA followed by Dunnett’s test. Error bars represent the mean ± SEM. *P < 0.05, **P < 0.01, ***P < 0.001, ****P < 0.0001. Abbreviations: ns, not significantly different; IEL, intraepithelial lymphocyte; SPL, splenocyte; PPs, Peyer’s patches.

The absolute number of CD8αβ^+^ IELs in TLR2-deficient, TLR4-deficient, and TLR9-deficient mice, and in mice deficient in all three TLRs, exhibited a similar reduction to that in Atb-treated mice. Although TLR2^−/−^ TLR4^−/−^ TLR9^−/−^ mice showed the most significant reduction in the absolute number of CD8αβ^+^ IELs (Figures 3A,B), no difference was observed in the number of CD8αβ^+^ SPLs in these TLR-deficient mice (Figure 3C). Moreover, the number of PPs and the length of small intestines showed no abnormality in TLR-deficient mice (Figures 3D,E). These data suggested that commensal bacteria maintained CD8αβ^+^ IELs indirectly *via* TLRs.

### Commensal Bacteria-Dependent CD8αβ^+^ IELs Exhibit Potent Microbicidal Activity

**Figure 4:**
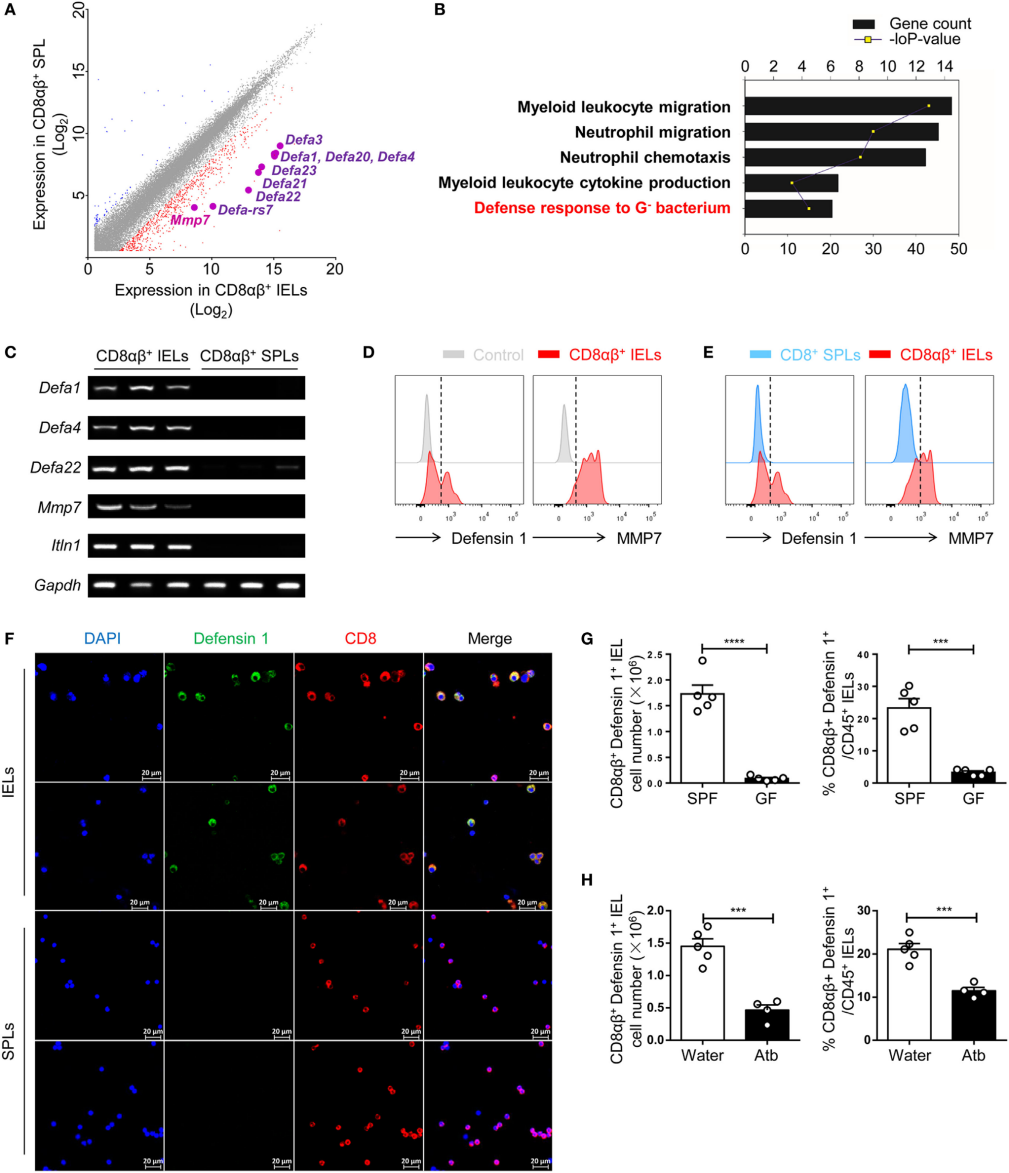
Expression of a series of genes that mediate the microbicidal response is upregulated in CD8αβ^+^ IELs. **(A)** CD45^+^ CD3^+^ CD8α^+^ CD8β^+^ TCRβ^+^ single cells were sorted from IELs and SPLs and subjected to microarray analysis. The difference in gene expression by microarray analysis is shown in a scatterplot and compares CD8αβ^+^ IELs and CD8αβ^+^ SPLs. Expression of several genes encoding α-defensins was upregulated in CD8αβ^+^ IELs and are highlighted. Two samples were analyzed per group with each sample sorted from 10 wild-type mice. **(B)** Gene ontology (GO) analysis of the genes upregulated in CD8αβ^+^ IELs. Five representative pathways were selected from the top 30 from GO enrichment. **(C)** The mRNA expression of *Defa1, Defa4, Defa22, Mmp7*, and *Itln1* in CD8αβ^+^ IELs and CD8αβ^+^ SPLs was measured by RT-PCR. Three samples were analyzed per group with each sample sorted from 10 wild-type mice. **(D,E)** Expression of defensin 1 and MMP7 in CD8αβ^+^ IELs (red histogram), control staining (gray histogram), and CD8αβ^+^ SPLs (blue histogram) detected by flow cytometry in gated CD45^+^CD3^+^CD8α^+^ CD8β^+^TCRβ^+^ single cells. The control in **(D)** (gray histogram) denoted that no primary antibody but only fluorescent secondary antibody had been added. Data are representative of three independent experiments. **(F)** IELs and purified CD8αβ^+^ SPLs were collected and the presence of defensin 1 (green) in CD8^+^ (red) IELs and SPLs detected by immunofluorescence. Data are representative of two independent experiments. **(G,H)** The absolute number and proportion of defensin 1^+^CD8αβ^+^ IELs were calculated and compared between GF and SPF mice **(G)** or water-treated mice and Atb-treated mice **(H)**, *n* = 4–5 mice per group. GF and SPF mice on a BALB/c background were used in **(G)**, but all the other mice used in this figure were on a C57BL/6 background. Data are representative of two independent experiments. Unpaired *t*-test. Error bars represent the mean ± SEM. ***P < 0.001, ****P < 0.0001. Abbreviations: IEL, intraepithelial lymphocyte; SPL, splenocyte; SPF, specific pathogen-free; GF, germ-free; Atb, antibiotic.

Our data demonstrated an essential role for commensal bacteria in the maintenance of CD8αβ^+^ IELs in the epithelium of the small intestine. Hence, questions arose: why do the number of cytotoxic CD8αβ^+^ IELs correlate with commensal bacteria and, during homeostasis do cytotoxic CD8αβ^+^ IELs have other functions with regards to the commensal bacteria within the small intestine? To gain insight into these questions, CD8αβ^+^ IELs (purity >97%) were compared with CD8αβ^+^ SPLs by microarray analysis (Figure S2C in Supplementary Material). Without stimulation *in vitro*, CD8αβ^+^ IELs exhibited a 20-fold increase in the expression of a series of genes that mediate antibacterial processes (e.g., *Defa1, RegIII*γ, and *lypd8*). At the transcriptional level, levels of the canonical effector molecule of CTLs, interferon (IFN)-γ, increased threefold in CD8αβ^+^ IELs relative to CD8αβ^+^ SPLs (Figure S2A in Supplementary Material). Expression of genes expressed specifically in Paneth cells and epithelial cells (e.g., *Sox 9, Ctnnb1, Ephb3, Epcam, Lyz 2, Lyz1*, and *Cd24a*) was not upregulated in CD8αβ^+^ IELs, demonstrating that IEL contamination by Paneth cells and epithelial cells was unlikely (Figure S2B in Supplementary Material). Among the genes whose expression was increased in CD8αβ^+^ IELs were those for a family of α-defensins. This included *mmp7*, a member of a class of genes encoding matrix metalloproteinases known to process inactivated mouse α-defensins to mature-active peptides (Figure 4A) (39). Gene ontology (GO) analyses revealed that CD8αβ^+^ IELs showed increased expression of the genes that mediate the defense response to Gram-negative bacteria (Figure 4B). RT-PCR of purified CD8αβ^+^ IELs and CD8αβ^+^ SPLs demonstrated expression of genes of the α-defensin family, including *mmp7*, in CD8αβ^+^ IELs (Figure 4C).

The presence of α-defensins in CD8αβ^+^ IELs was assessed by flow cytometry and immunofluorescence. Correspondingly, defensin 1 and mmp7 were detected in CD8αβ^+^ IELs but not in CD8αβ^+^ SPLs (Figures 4D,E). It is difficult to distinguish α-defensins in CD8αβ^+^ IELs from α-defensins in Paneth cells *in situ*, so purified CD8αβ^+^ IELs were assessed by immunofluorescence, and defensin 1-positive granules were detected (Figure 4F). CD8αβ^+^ IELs have a “resident effector memory” phenotype in that IFN-γ, granzyme B, tumor necrosis factor-α, CD44, CD69, CD103, and CD5 are detected readily (Figure S3 in Supplementary Material), as described previously (2, 5, 40). In GF- and Atb-treated mice, there was a decrease in the number of defensin 1^+^ CD8αβ^+^ IELs in comparison with untreated SPF mice, indicating that these defensin 1^+^ CD8αβ^+^ IELs were dependent upon commensal bacteria (Figures 4G,H). In summary, CD8αβ^+^ IELs contained AMPs and antimicrobial peptide-carrying CD8αβ^+^ IELs showed a positive relationship with commensal bacteria.

### Commensal Bacteria-Dependent CD8αβ^+^ IELs Can Inhibit the Growth of Bacteria Directly

We had shown expression of α-defensins in CD8αβ^+^ IELs. Defensins are broad-spectrum AMPs that act against various bacteria (23). Hence, next we assessed the antimicrobial activity of CD8αβ^+^ IELs *ex vivo*. As mentioned above, Paneth cells are thought to be the only source of α-defensins in the mouse intestine. Also, the antimicrobial activity of Paneth cells by secretion of microbicidal α-defensins (also called cryptdins) was assessed by the co-culture of isolated crypt secretions on bacteria (41). Based on that study, we conducted a co-culture experiment with CD8αβ^+^ IELs and *E. coli*.

**Figure 5:**
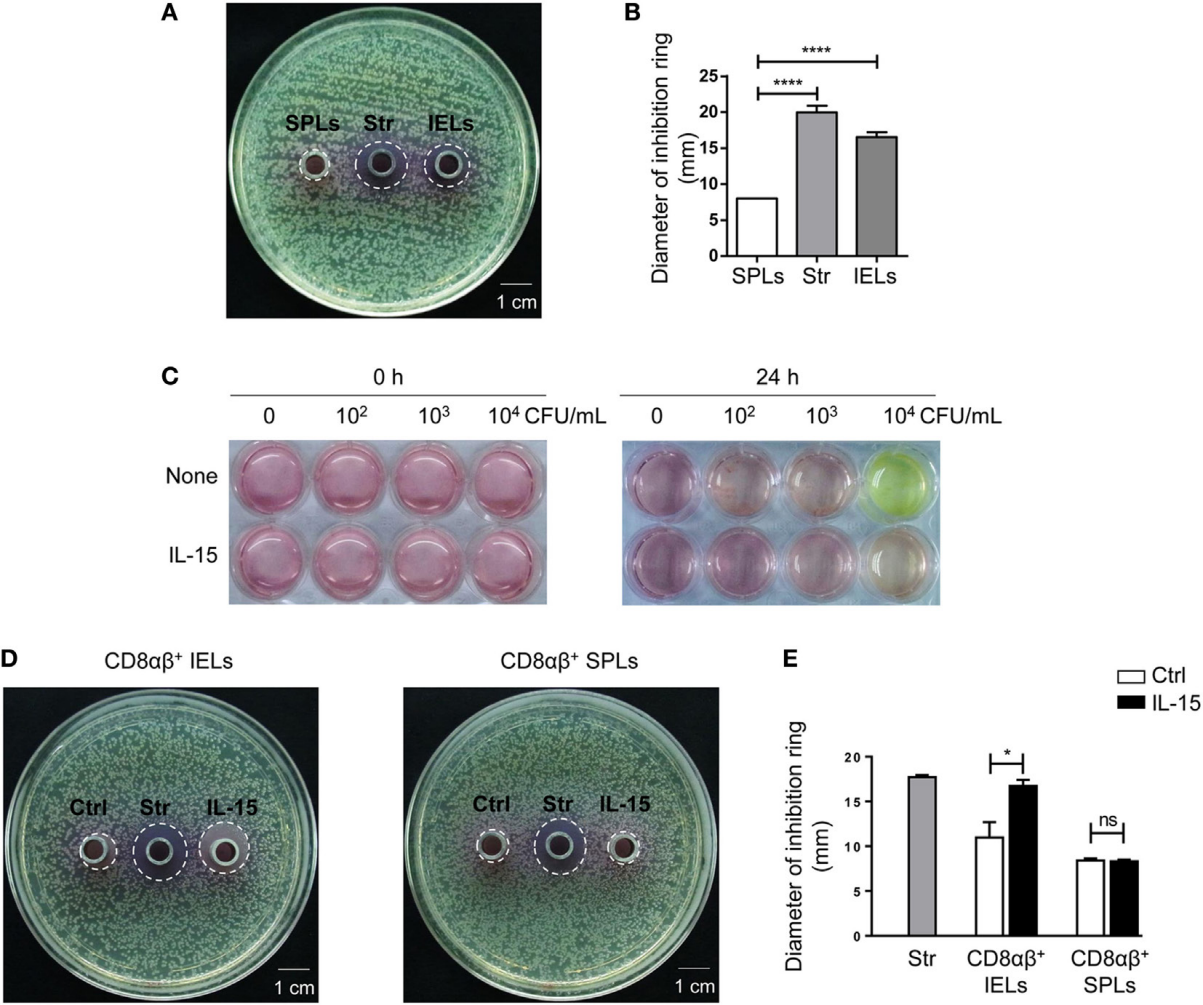
The supernatant from CD8αβ^+^ IELs can inhibit the formation of bacterial colonies directly. **(A,B)** CD8αβ^+^ IELs and CD8αβ^+^ SPLs were sorted and cultured for 24 h, with activation by PMA and ionomycin during the final 6 h. After 24 h, the supernatants were collected for an antimicrobial-activity assay. Iscove’s modified Dulbecco’s medium (IMDM) with streptomycin (0.1 mg/mL) was added to the middle Oxford cup as a positive control (labeled as “Str,” middle). Supernatants from CD8αβ^+^ SPLs (labeled as “SPLs,” left) and CD8αβ^+^ IELs (labeled as “IELs,” right) were added into Oxford cups, respectively, on the left and right side of the agar plate **(A)**. Inhibition rings were observed after 18 h. The diameter of the inhibition rings was measured and is shown in **(B)**. One-way ANOVA followed by Dunnett’s test. Each cell sample was sorted from 5 wild-type mice. Data are representative of three independent experiments; error bars represent the mean ± SEM. ****P < 0.0001. **(C)** IELs (1 × 10^6^ cells) and increasing concentrations of *Escherichia coli* (CFU/mL), indicated in the Figure, were co-cultured with or without IL-15. The color of the medium indicated the acidity produced by bacterial proliferation. Data are representative of three independent experiments. **(D,E)** CD8αβ^+^ IELs were sorted and cultured with or without the IL-15/IL-15R complex for 24 h, after which the supernatants were collected for an antimicrobial-activity assay. IMDM with streptomycin (0.1 mg/mL) was added to the middle Oxford cup as a positive control (labeled as “Str,” middle). Oxford cups containing supernatants from CD8αβ^+^ IELs activated without (labeled as “Ctrl,” left) or with (labeled as “IL-15,” right) the IL-15/IL-15R complex were located, respectively, on the left and right side of the agar plate **(D)**. Inhibition rings were observed after 18 h. The diameter of the inhibition rings was measured and is shown in **(E)**. Unpaired *t*-test. Each cell sample was sorted from 5 wild-type mice. Data are representative of two independent experiments. Error bars represent the mean ± SEM. *P < 0.05. Abbreviations: ns, not significantly different; IEL, intraepithelial lymphocyte; SPL, splenocyte; Str, streptomycin.

Sorted CD8αβ^+^ IELs and CD8αβ^+^ SPLs were cultured for 24 h, and activated with PMA and ionomycin for the final 6 h, after which supernatants were collected, and antimicrobial activity determined. As described in Section “Materials and Methods,” petri plates containing LB-Agar medium were coated with *E. coli* and then Oxford cups were placed on the plates and filled with supernatants. The petri plates were transferred into an incubator at 37°C, and bacterial colonies and inhibition rings were observed 18 h later. No inhibition rings were observed around Oxford cups filled with supernatants from purified CD8αβ^+^ SPLs, but inhibition rings were observed around Oxford cups filled with supernatants from purified CD8αβ^+^ IELs (Figure S2D in Supplementary Material; Figure 5A,B). These data showed that secretion from stimulated CD8αβ^+^ IELs could inhibit the growth of bacteria, likely due to AMPs such as defensins.

Interleukin-15 has been reported to influence the activation and proliferation of small-intestinal TCRγδ^+^ IELs as well as peripheral CD8^+^ T cells (42–46). Hence, the effect of IL-15 on the secretion of AMPs by CD8αβ^+^ IELs was assessed. *E. coli* was cultured with IELs and bacterial growth was judged by the color of the culture medium. Culture medium with IELs and 10^4^ CFU of *E. coli* turned yellow after 24 h, whereas the culture medium with IELs and 10^4^ CFU of *E. coli* with added IL-15 did not turn yellow (Figure 5C). According to the antimicrobial assay described above, supernatants from purified CD8αβ^+^ IELs stimulated with IL-15 also produced inhibition rings (Figures 5D,E). These data suggested that IL-15 promoted the antimicrobial activity of CD8αβ^+^ IELs.

## Discussion

The intestinal epithelium serves as a primary barrier between the host and a large number of bacteria in the lumen of the small intestine. A large proportion of IELs at this site are CD8αβ^+^. However, as CD8αβ^+^ IELs are closely correlated with commensal bacteria, their function besides cytotoxicity is unclear. Nor is their role in homeostasis established. Herein, commensal bacteria were demonstrated to regulate the number of CD8αβ^+^ IELs along the intestinal epithelium. Also, the importance of TLRs in this process was shown. Microarray analysis identified a series of genes that encode antibacterial peptides in commensal-dependent CD8αβ^+^ IELs. The supernatants of these IELs were found to have direct antibacterial activity. These results suggest that commensal-dependent CD8αβ^+^ IELs can inhibit the growth of bacteria effectively.

MyD88 signaling has been reported to induce IL-15 production from IECs and, in addition, to induce the accumulation of TCRγδ^+^CD8αα^+^ and TCRαβ^+^CD8αα^+^ IELs (42). CD8αβ^+^ IELs are located in the same microenvironment, so they may also be regulated by IL-15 induced *via* TLR-Myd88 signaling. CD8αβ^+^ IELs do not express TLRs, so it is possible that a cytokine (e.g., IL-15) may mediate the effect of commensal bacteria on CD8αβ^+^ IELs, because lower expression of IL-15 in GF mice in comparison with SPF mice was observed (data not shown). IECs, intestinal macrophages, and DCs express IL-15, and macrophages from gut microbiota-depleted mice show lower expression of IL-15 (47). We demonstrated that the IL-15/IL-15R complex can enhance the antimicrobial activity of CD8αβ^+^ IELs *in vitro* (Figures 5D,E). These data suggested that IL-15 expression was influenced by commensal bacteria, and raised the possibility that IL-15 may mediate the effect between commensal bacteria and CD8αβ^+^ IELs.

Analyses of the 16S rDNA of Atb-free mice and Atb-treated mice in this study suggested that CD8αβ^+^ IELs may be influenced by specific groups of bacteria. It was reported recently that *Lactobacillus reuteri*, a probiotic bacterium whose numbers were decreased dramatically by the antibiotics in their study, induced CD4^+^CD8αα^+^ T cells in the intestinal epithelium (48). That recent report raised the possibility that particular bacterial species may induce specific subsets of IELs because they are located in a similar microenvironment. Our microbiota-transplantation experiment showed that *Bifidobacterium* species, removed by antibiotics in our study, could induce an increase in the number of CD8αβ^+^ IELs effectively. How these bacteria influence CD8αβ^+^ IELs or other IEL subsets at the mechanistic level is not known.

Comparative microarray analysis of CD8αβ^+^ IELs and CD8αβ^+^ SPLs cells showed that expression of a series of genes encoding AMPs (especially α-defensins) was upregulated in CD8αβ^+^ IELs but not SPLs. Studies have suggested that Paneth cells are the only source of α-defensins in mice (21–23). CD8αβ^+^ T cells in long-term HIV-1 non-progressors were demonstrated to secrete α-defensin 1, 2, and 3 and suppress HIV-1 replication. In that study, CD8αβ^+^ T cells did not express mRNA for the defensins but rather acquired α-defensin protein by taking up defensins secreted originally by neutrophils (49–51). It has also been reported that, in some cases, CD8^+^ T cells among peripheral-blood lymphocytes from patients with severe cutaneous reactions can upregulate expression of α-defensin 1–3, and that this correlates with disease severity (52). These findings suggest that the function of CD8^+^ T cells can be expanded under specific physiologic conditions. In our study, a series of α-defensins were found specifically in CD8αβ^+^ IELs at gene and protein levels in homeostasis. Furthermore, supernatants from purified CD8αβ^+^ IELs inhibited the growth of bacteria. Taken together, these data demonstrate murine CD8αβ^+^ IELs to be producers of α-defensins in the intestine.

α-defensins are broad-spectrum microbicides that are major determinants of intestinal micro-ecology (53). For many years, Paneth cells were thought to be the only source of α-defensins and, as such, were considered important in host–microorganism homeostasis. Paneth cells are located at the base of crypts between villi. As a result, secreted bactericidal peptides are released into the lumen from the base of the crypts, with maximal antimicrobial activity at the bottom of the crypt, but a decreased concentration and activity in the middle and at the top of the villi. In this study, CD8αβ^+^ IELs distributed along the epithelial layer of the small intestine were demonstrated to secrete α-defensins, which may be important as supplements to the α-defensins produced by Paneth cells. Together, CD8αβ^+^ IELs and Paneth cells may combine effectively to prevent bacterial invasion. This hypothesis is consistent with the concept that CD8αβ^+^ IELs and the microbiota regulate each other reciprocally.

Interestingly, investigation of another subset of IELs in the intestinal epithelium, TCRγδ^+^ IELs, revealed that the latter could secrete RegIIIγ and RegIIIβ, which are antimicrobial factors of the small intestine known to protect against invading resident bacteria (54). Taken together with the data herein, those findings suggest that the intestinal epithelium is a specialized microenvironment that serves as a physical and immune barrier to bacteria at the mucosal interface. Lymphocytes at this surface may help control pathogenic and resident bacteria to maintain host–microbial homeostasis. It is possible that the production of AMPs could also be found in T cells located in other mucosal tissues.

There may be some key factors in this microenvironment that induce the production of defensins and other AMPs by IELs. The data herein suggest that IL-15 may be a candidate, and this issue needs more investigation. In conclusion, this study demonstrated a close relationship between commensal bacteria and CD8αβ^+^ IELs. It uncovered a new function for CD8αβ^+^ IELs in the intestinal epithelium, and implies that lymphocytes in certain special microenvironments may gain new functions. Exploration of these cells in differing microenvironments may provide new insights into treatment for bacterial-based diseases and disorders.

## Ethics Statement

All of the animal protocols were approved by Local Ethics Committee for Animal Care and Use at University of Science and Technology of China (USTCACUC1601007). The sample size was determined by the “resource equation” method.

## Author Contributions

BC designed, carried out and analyzed the experiments, and wrote the manuscript. XN analyzed part of the data. RS established methods for flow cytometry, supervised the experiments, and revised the manuscript. BZ and HongW provided GF mice. ZT and HaimingW provided the study strategy, supervised research, and revised the manuscript.

## Conflict of Interest Statement

The authors declare that the present study was conducted in the absence of commercial or financial relationships that could be construed as a potential conflict of interest.
