# Supplementary material for: Commensal Bacteria-Dependent CD8αβ+ T Cells in the Intestinal Epithelium Produce Antimicrobial Peptides
Source: Front Immunol. 2018 May 16;9:1065. doi: 10.3389/fimmu.2018.01065 (PMC5964211; doi:10.3389/fimmu.2018.01065)
Supplement: Supplementary file 1 [file Data_Sheet_1.PDF]

Supplementary Material

Commensal bacteria-dependent CD8αβ^{+} T cells in the intestinal epithelium produce antimicrobial peptides

Banru Chen[1], Xiang Ni[1], Rui Sun[1, 2], Benhua Zeng[3], Hong Wei[3 *], Zhigang Tian[1, 2 *], Haiming Wei[1, 2 *]

[1]Institute of Immunology and the CAS Key Laboratory of Innate Immunity and Chronic Disease, School of Life Sciences and Medical Center, University of Science and Technology of China, Hefei City, Anhui, China
[2] Hefei National Laboratory for Physical Sciences at Microscale, University of Science and Technology of China, Hefei City, Anhui, China
[3] Department of Laboratory Animal Science, College of Basic Medical Sciences, Army Medical University, Chongqing, China.

Correspondence*:

Haiming Wei
ustcwhm@ustc.edu.cn

Zhigang Tian
tzg@ustc.edu.cn

Hong Wei
weihong63528@163.com

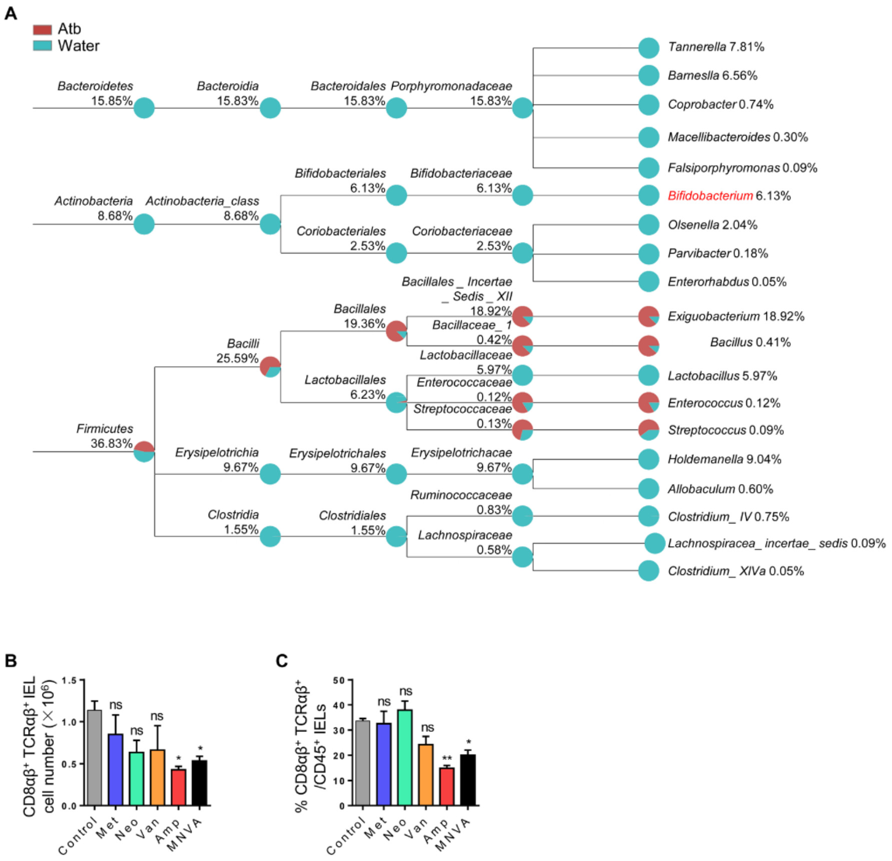

Supplementary Figure 1. CD8αβ$^+$ IELs show higher sensitivity to ampicillin treatment. (A) Bacterial composition included in the phyla *Bacteroidetes*, *Actinobacteria* and *Firmicutes* at the genus level, *Bifidobacterium* is highlighted in red. n = 3 mice per group. (B and C) Three-week-old SPF mice were fed with normal water, water containing a combination of antibiotics, or water containing each antibiotic alone (Amp = ampicillin; Van = vancomycin; Neo = neomycin; Met = metronidazole) for 5 weeks. The absolute number (B) and percentage (C) of CD8αβ$^+$ IELs from the indicated mice were analyzed by flow cytometry, n = 3–6 mice per group. One-way ANOVA followed by Dunnett's test. All mice used were on a C57BL/6 background. Data are representative of two independent experiments. Error bars represent the mean ± SEM. *P < 0.05, **P < 0.01; ns, not significantly different. IEL, intraepithelial lymphocyte. Data are representative of two independent experiments. Error bars represent the mean ± SEM. *P < 0.05, ***P < 0.001, ****P < 0.0001; ns, not significantly different. IEL, intraepithelial lymphocyte; Atb, antibiotic.

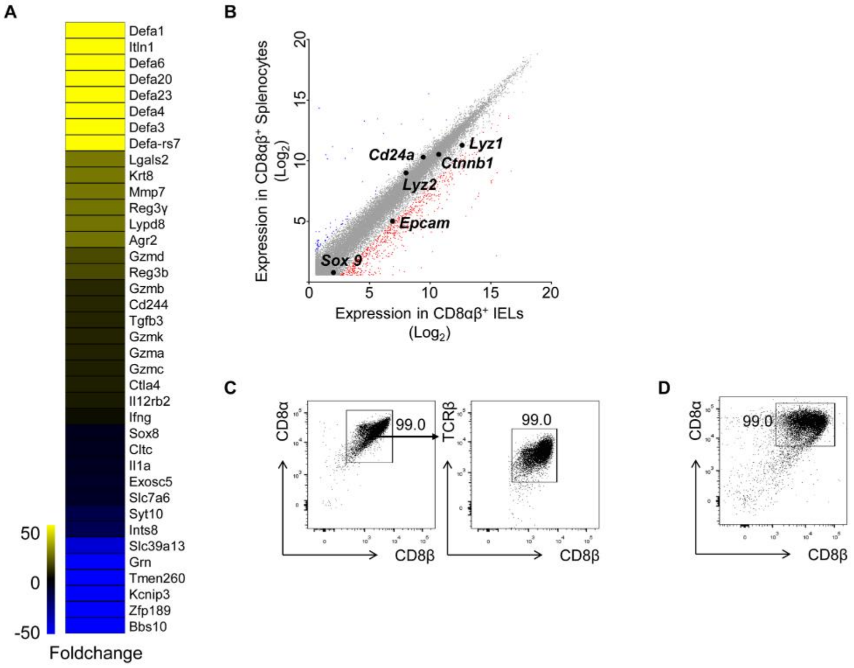

Supplementary Figure 2. High-purity CD8αβ^{+} IELs were used for microarray analysis and bactericidal assay. (A) CD45^{+} CD3^{+} CD8β^{+} CD8α^{+} TCRβ^{+} single cells were sorted from IELs and SPLs and subjected to microarray analysis. Expression of the canonical genes upregulated or downregulated in CD8αβ^{+} IELs relative to CD8αβ^{+} SPLs are shown. Three samples were analyzed per group and with each sample sorted from 10 wild-type mice. (B) The difference in gene expression by microarray analysis is shown in a scatterplot defined in Figure 4A comparing CD8αβ^{+} IELs and CD8αβ^{+} SPLs. Several genes expressed by Paneth cells are highlighted. (C and D) Purity of CD8αβ^{+} IELs sorted by flow cytometry (A) and magnetic-activated cell sorting (MACS) (B) are shown. Each cell sample was sorted from 10 wild-type mice. IEL, intraepithelial lymphocyte; SPL, splenocyte.

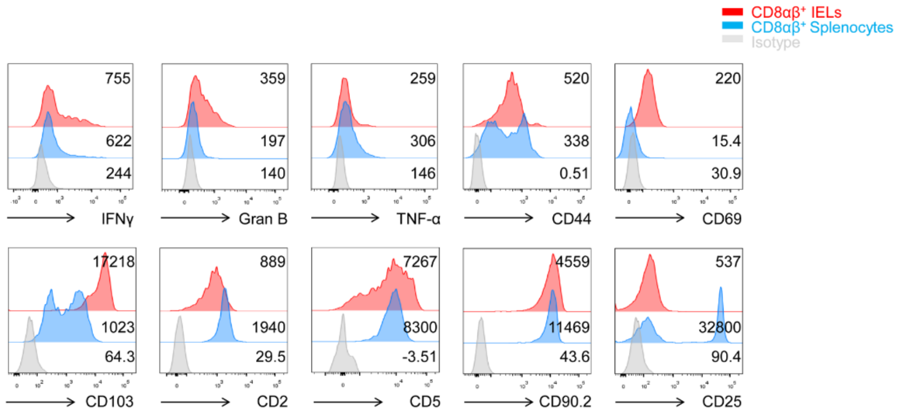

Supplementary Figure 3. CD8αβ$^+$ IELs show a "resident effector memory" phenotype. Expression of indicated markers in CD45$^+$ CD3$^+$ CD8β$^+$ CD8α$^+$ IELs and SPLs was analyzed by flow cytometry. Mean fluorescence intensity (MFI) is shown. Data are representative two independent experiments.

Supplementary Table 1. Antibodies for flow cytometry and magnetic-activated cell sorting.

Fluorescence	Antigen	Clone	Company	Catalog number	Isotype control	Dilution
FITC	CD8β	H35-17.2	eBioscience	11-0083	Rat IgG2b, κ	1:200
FITC	CD69	H1.2F3	BD	553236	ArH IgG1, λ_3	1:200
488	CD90.2	30-H12	Biolegend	105315	Rat IgG2b, κ	1:200
PE	CD8α	53-6.7	BD	553032	RatIgG2a, k	1:200
PE	CD8β	H35-17.2	BD	550798	Rat IgG2b,κ	1:200
PE	CD103	M290	Biolegend	121406	Rat IgG2a,κ	1:200
PE	CD2	RM2-5	BD	553112	Rat IgG2b, λ	1:200
PE	CD25	PC61	BD	553866	Rat IgG1,λ	1:200
Percp-CY5.5	CD3e	145-2C11	Biolegend	100328	AH IgG	1:200
Percp-CY5.5	CD45.2	104	Biolegend	109828	Ms IgG2a,κ	1:200
Percp-CY5.5	CD44	IM7	BD	560570	Rat IgG2b,k	1:200
PE-CY7	CD45	30-F11	Biolegend	103114	Rat IgG2b,k	1:200
PE-CY7	IFN-γ	XMG1.2	eBioscience	557649	Rat IgG1,κ	1:200
APC	TCR β	H57-597	Biolegend	109212	ArH IgG	1:200
APC	CD8α	53-6.7	Biolegend	100712	Rat IgG2a,k	1:200
APC	CD5	53-7.3	BD	100625	Rat IgG2a, κ	1:200
APC-CY7	CD3e	145-2C11	Biolegend	100330	AH IgG	1:200
Purified	α-Defensin 1	Polyclonal	Abcam	ab122884	IgG	1:100
Purified	MMP7	Polyclonal	Abcam	ab4044	IgG	1:100
Purified	CD8	53-6.7	Biolegend	100702	Rat IgG2a.κ	1:200
FITC	Donkey Anti-Goat IgG	Polyclonal	Santa Crus	sc-2024	-	1:200
FITC	Donkey Anti-Rabbit IgG H&L	Polyclonal	Abcam	ab97050	IgG	1:200
488	Donkey Anti-Goat IgG H&L	Polyclonal	Abcam	ab150129	IgG	1:500
Alexa 594	Donkey Anti-Rabbit IgG H&L	Polyclonal	Abcam	ab150076	IgG	1:100
Biotin	CD8β	YTS156.7.7	Abcam	126604	Rat IgG2b, κ	1:400

Supplementary Table 2. Primers for RT-PCR.

Gene	Forward (5’-3’)	Reverse (5’-3’)
Defa1	CCTGCTCATCCTAATCCATCCA	CGTTCTCTTCCTTTGCAGCCT
Defa4	CACTTGTCCTCCTCTCTGCC	GTCATCTGCATGTTCAGCGG
Defa22	AATCCTCCAGGTGACTCCCA	CTCGACAATTTTTCATGAAGAGCA
Mmp7	CTCACCCTGTTCTGCTTTGTG	TGATCCACTACGATCCGAGGT
Itln1	ACTGAACATCACTGCATCGGT	GAGAAGTCAGGGCCAATCCC
Gapdh	TGCACCACCAACTGCTTAG	GGATGCAGGGATGATGTTC
